# Effects of Acute Cold Stress after Intermittent Cold Stimulation on Immune-Related Molecules, Intestinal Barrier Genes, and Heat Shock Proteins in Broiler Ileum

**DOI:** 10.3390/ani12233260

**Published:** 2022-11-23

**Authors:** Xiaotao Liu, Shuang Li, Ning Zhao, Lu Xing, Rixin Gong, Tingting Li, Shijie Zhang, Jianhong Li, Jun Bao

**Affiliations:** 1 College of Life Science, Northeast Agricultural University, Harbin 150030, China; 2 College of Animal Science and Technology, Northeast Agricultural University, Harbin 150030, China

**Keywords:** broiler ileum, cold stimulation, immunoglobulins, cytokines, toll-like receptors, heat shock proteins

## Abstract

**Simple Summary:**

Animal welfare and health will be negatively impacted by cold stress, resulting in decreased production performance, immune imbalance, and decreased antioxidant capacity. Previous studies focused more on the side effects of low temperature, but animals have the ability to adapt to the environment by regulating metabolic and endocrine processes. The objective of this study was to compare cold resistant of broilers with and without cold stimulation training. By detecting the changes in immunoglobulins, cytokines, toll-like receptors, gut barrier genes, and heat shock proteins gene expression levels before and after acute cold stress, the optimal cold training method was finally determined. The results of our study show that cold stimulation training for 6 h with an interval of one day, at 3 °C lower than the conventional temperature, can change the immune function of broilers. This cold stimulation can also lessen the intestinal damage when subjected to acute cold stress. This research offers a theoretical foundation for the regulation of immune function in broilers raised in cold environments, as well as a scientific basis for the development of cold adaptation in broilers.

**Abstract:**

Cold stress will have a negative impact on animal welfare and health. In order to explore the effect of intermittent cold stimulation training on the cold resistance of broilers. Immune-related and intestinal barrier genes were detected before and after acute cold stress (ACS), aiming to find an optimal cold stimulation training method. A total of 240 1-day-old Ross broilers (*Gallus*) were divided into three groups (G1, G2, and G3), each with 5 replicates (16 chickens each replicate). The broilers of G1 were raised at normal temperature, while the broilers of G2 and G3 were treated with cold stimulation at 3 °C lower than the G1 for 3 h and 6 h from 15 to 35 d, respectively, at one-day intervals. At 50 d, the ambient temperature for all groups was reduced to 10 °C for six hours. The results demonstrated that before ACS, *IL6*, *IL17*, *TLR21*, and *HSP40* mRNA levels in G3 were apparently down-regulated (*p* < 0.05), while *IL8* and *Claudin-1* mRNA levels were significantly up-regulated compared with G1 (*p* < 0.05). After ACS, *IL2*, *IL6*, and *IL8* expression levels in G3 were lower than those in G2 (*p* < 0.05). Compared to G2, *Claudin-1*, *HSP90* mRNA levels, *HSP40*, and *HSP70* protein levels were increased in G3 (*p* < 0.05). The mRNA levels of *TLR5*, *Mucin2*, and *Claudin-1* in G2 and *IL6*, *IL8*, and *TLR4* in G3 were down-regulated after ACS, while *IL2*, *IL6*, and *IL17* mRNA levels in G2 and *HSP40* protein levels in G3 were up-regulated after ACS (*p* < 0.05). Comprehensive investigation shows that cold stimulation at 3 °C lower than the normal feeding temperature for six hours at one day intervals can enhanced immune function and maintain the stability of intestinal barrier function to lessen the adverse effects on ACS in broilers.

## 1. Introduction

Cold is the most common stress factor for livestock and poultry in northern alpine regions [1]. For broilers (*Gallus*), exposure to a sudden drop in the ambient temperature of more than 10 °C or prolonged exposure to temperatures that are more than 4 °C lower than the ambient temperature will result in cold stress, which will have an impact on health and welfare of the birds [2] and lead to immune dysfunction [3] and physiological disorders [4,5]. Previous studies [4,6] have focused mostly on the side effects of exposure to low temperatures, but animals have the ability to adapt to their environment. Therefore, the present priority is to explore a training method to establish cold acclimation in poultry. After repeated cold stimulation, broilers can establish cold adaptation by regulating endocrine and metabolic processes and improve the ability of resist disease and cold stress [7,8]. The establishment of cold adaptation can, therefore, be measured by immune indices [9,10,11]. Immune indices include immunoglobulins, cytokines, and toll-like receptors. Immunoglobulin is a highly effective antibody. The initiation and regulation of the inflammatory response are significantly influenced by cytokines. The host can be protected from pathogen invasion by toll-like receptors. Su et al. [12] demonstrated that when broilers were trained with cold stimulation, the ileum structure was complete and the level of proinflammatory cytokines was reduced when broilers were subjected to 7 °C (24 h) ACS. Therefore, when broilers have thermoregulation ability, cold training for broilers is critical to establishing cold adaptation. Further explore strategies to improve the anti-stress and immune abilities of broilers.

To cope with various challenges, the body will activate the protein control system or cell death signaling pathway to produce an efficient stress response [13]. Immunoglobulins refer to proteins with antibody activity [14]. The mRNA level of immunoglobulin can reflect the strength of immune function. *IgA* performs a key function in protecting the gut against microbial invasion [15]. *IgG* has a range of biological activities, such as antiviral and anti-exotoxin. Studies have shown that broilers with colitis have lower levels of *IgA* and *IgG* in the ileum [16,17,18]. Olfati et al. The authors of [19] raised 22 d old broilers in a cold environment (12 °C) until they reached 42 d and noticed that cold stress decreased substantially serum *IgG* levels. Severe cold stress will reduce the expression level of immunoglobulins in animals, causing damage to the body. Early cold training has been shown by Su et al. [7] to up-regulate the intestinal *IgA* level in broilers and enhance broilers resistance to cold stress. It, therefore, follows that the body’s immune function can be strengthened by the appropriate cold stimulation.

Cytokines are protein molecules produced by immune cells and related cells to regulate cellular functions and participate in anti-inflammatory and pro-inflammatory processes [8,20]. According to studies, prolonged cold stimulation in broilers’ ileums can raise *IL-4* levels and lower *IFN-γ* levels, causing an immunological imbalance [21]. *IL-8* causes local inflammation in the body by stimulating leukocytes. *IFN-γ* can boost immune response capability by activating the activity of immune-related cell. Perelman et al. [22] reported that the levels of *IL-1β, IL-8*, and *TNF-α* in bronchial tissues of patients with cold airway hyperresponsiveness increased significantly after acute cold stress, resulting in a significant inflammatory response. *IL-6* can identify pathogen signals [20]. Yildirim et al. [23] found that the level of *IL-6* in liver tissues of rats exposed to a low temperature at 10 °C was significantly up-regulated, while Vargovic et al. [24] demonstrated that raising rats in a 4 °C cold environment for seven consecutive days reduced the expression level of *IL-6* in liver and lung tissue. Thus, stress can change immune function by regulating the secretion of cytokines.

Toll-like receptors not only mediate defense against the invasion of microbial pathogens, but also regulate immune homeostasis [25]. When toll-like receptors recognize pathogenic molecules, they can activate downstream signaling pathways (NF-κB) and induce the production of cytokines to participate in the immune response [26,27]. *TLR2* and *TLR4* can bind to the LPS of gram-negative bacteria and directly induce the expression of inflammatory cytokines [28]. *TLR5* can recognize the signal from the flagellum, which causes the production of proinflammatory factors. *TLR4* and *TLR5* levels in the mesenteric lymph nodes of Malabari goats were dramatically reduced, while the *TLR2* level was considerably elevated after five days of continuous exposure (six hours per day) at 27–34 °C in the summer [29]. Li et al. [30] discovered that the mRNA level of *TLR7* in the duodenum of broilers was substantially higher after cold stimulation training (43 d/3 °C lower than the control). *TLR7* is responsible for identifying the single-stranded RNA of viruses invading the body, which is crucial for defending the body against viral infection [31].

The intestinal barrier is a protective barrier that prevents pathogens and toxic compounds from passing through the systemic circulation [32]. The absence of a tight junction complex will reduce barrier protection and thus will affect intestinal permeability [33]. Studies have shown that markers for evaluating the health of the intestinal barrier can be *Claudin-1, Occludin*, and *ZO-1* mRNA levels [34,35,36]. *Mucin2* produced by goblet cells can protect the intestine from invasion by bacteria [37], has an antibacterial effect [38]. Uerlings et al. [39] reported that the levels of *Claudin-1* and *Occludin* in broilers’ jejunum exposed to high temperatures for 24 h were significantly elevated. Some studies proved that long-term chronic stress can reduce the levels of tight junction proteins, impair intestinal barrier function, and increase permeability [40,41]. Therefore, whether early appropriate cold stimulation training can mitigate the adverse impacts of late ACS on the gut by changing the intestinal structure needs further study.

Heat shock proteins (HSPs) are vital elements in the regulation of the stress response and function to protect the integrity of epithelial cells and alleviate stress injury because they act as molecular chaperones [42]. Under normal physiological conditions, HSPs are maintained at low levels. When an organism encounters sudden environmental temperature changes, a large number of HSPs will be produced to enhance resistance to stress injury [43]. *HSP40* is a cooperative protein of *HSP70*, participating in the dissociation and transmembrane transport of proteins. The high expression level of *HSP70* can increase tolerance to various stressors, greatly improving the survival rate of the organism under stress [44]. Zaglool et al. [45] proved that *HSP90* level in broilers was rapidly up-regulated under acute heat stress at 36 °C for six hours. Wei et al. [20] showed that the lower *HSP90* levels in broilers’ duodenum under acute cold stress and that cold stress altered immune function. In conclusion, the expression level of HSPs can be utilized as a stress response biomarker in animals.

The negative impacts of low temperatures on animals have earlier received more attention, but animals have the ability to adapt to the environment by regulating metabolic and endocrine processes [20,21]. Therefore, without affecting production performance, it is particularly important to explore methods of inducing cold adaptation in animals to enable them to resist cold stress. A comparative experiment was used in this study. The objective was to investigate the effects of ACS on immunity and resistance of broilers to cold stress. By analyzing the levels of gut immune-related and intestinal barrier genes in broilers before and after ACS, the optimal cold stimulation training program was defined which can improve cold resistance in broilers. The research can provide a theoretical foundation for the regulation of immune function of broilers in cold environments, and provide a scientific basis for the development of cold adaptation in broilers.

## 2. Materials and Methods

### 2.1. Animal Care and Experimental Design

**Figure 1:**
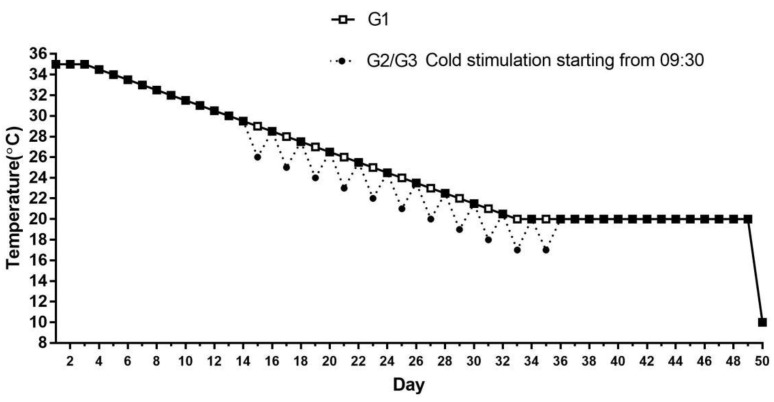
The specific experimental temperature scheme.

All experiments and methods utilized in the current research were conducted with the support of the Institutional Committee for Animal Care and Use of the Northeast Agricultural University in Harbin, China (IACUCNEAU20150616). A comparative experiment was used in this research; 240 Ross 308 broilers (Hongyan breeding and planting Park, Harbin, China), 1-day-old, were divided into 3 groups including the one group without cold stimulation training (cold stimulation for 0 h, G1) and 2 groups with cold stimulation training (cold stimulation for 3 h, G2 and cold stimulation for 6 h, G3), 5 replicates per group, 16 chickens per replicate. Broilers were raised in three climate chambers, and food and water were freely available at all times. Broilers were fed the entire starter diet from 1 to 21 d, containing 12.10 MJ/kg of metabolizable energy and 21.00% crude protein. Broilers were raised a grower diet at 22 d (19.00% CP and 12.80 MJ/kg ME) and maintained for 3 weeks. Broilers were raised on a finishing diet from 43 to 50 days of age, including 17.50% of CP and 13.20 MJ/kg of ME. The diets consist of corn, vitamin A, calcium chloride, Soybean meal, bran, sodium chloride, and vitamin D3 (Baishicheng, Harbin, China). Lighting regime: 1–3 d, 24 L:0 D; 4–50 d, 23 L:1 D. The humidity was kept between 60% and 70% for the first 14 days and 40–50% from 15 to 50 days of age. The specific experimental temperature scheme is shown in Figure 1. The G1 was managed according to the standard feeding temperature during the growth stage of broilers. Broilers of G2 and G3 were raised at 3 °C lower than G1, and the duration of cold stimulation was 3 h (9:30 a.m.–12:30 p.m.) and 6 h (9:30 a.m.–15:30 p.m.) at one day intervals from 15 to 35 days, respectively. All broilers were raised at 20 °C for 36–49 d. At 50 d, all broilers were suffered from ACS (10 °C) for 6 h (8:00 a.m.–14:00 p.m.).

One broiler was selected from each replicate group and put to death at 08:00 a.m. (pre-ACS) and 14:00 p.m. (ACS) on day 50, sections of the ileum from the broilers were cut, cleaned with normal saline, put them into liquid nitrogen instantly, and only then stored at −80 °C.

### 2.2. RNA Extraction and Reverse Transcription

TRIzol (Takara, Japan) was utilized to extract total RNA from broiler ileum tissue samples in accordance with the manufacturer’s instructions. The RNA was redissolved in 50 μL of enzyme-free water. Next, 1.5% agarose gel electrophoresis was used to determine the integrity of the RNA. The purity of the RNA was determined by measuring the OD260/OD280 ratio with a UV-spectrophotometer (Thermo Fisher Scientific, Carlsbad, CA, USA). Complementary DNA (cDNA) was synthesized by reverse transcription of RNA with ReverTra Ace qPCR RT Master Mix and gDNA Remover (Toyobo, Osaka, Japan), and then stored at −20 °C.

### 2.3. Quantitative Real-Time PCR Analysis

Sangon Bioengineering (Sangon Biotechnology, Shanghai, China) Co. Ltd. designed and synthesized the primers listed in Table S1. Real-time quantitative PCR was conducted using the LightCycler 480 II instrument (Roche, Switzerland). The total system consisted of 5 μL SYBR Green I, 3.4 μL enzyme-free water, 1 μL cDNA template, and 0.3 μL forward and reverse primers, respectively. qPCR program setup as: pre-denaturation at 95 °C for 60 s, repeated 40 cycles of denaturation at 95 °C for 15 s and then extension at 60 °C for 60 s. Each qPCR product was specific and its melting curve was unimodal. The 2^−∆∆CT^ method was used to calculate the mRNA levels of target genes, with the house-keeping gene β-actin as internal reference.

### 2.4. Western Blot Analysis

Western IP cell lysis solution (SparkJade, Harbin, China) containing 1% PMSF (SparkJade, Harbin, China) was used to extract total proteins from frozen broiler ileum tissue samples. The concentration of protein was quantified with Bicinchonininc Acid (BCA) protein concentration detection kit (SparkJade, Harbin, China) and adjusted to a uniform concentration (5 μg/μL). The same amounts of total protein (28 μg/condition) were placed on 12.5% gel (SparkJade, Harbin, China) for SDS-PAGE. The proteins were transferred to the NC membrane (SparkJade, Harbin, China) using semi-dry transfer equipment (Amersham Biosciences, Boston, MA, USA). Next, 5% skim milk 37 °C was sealed for 2 h and cleaned 3 times with PBST. Following that, the particular primary antibodies HSP40 and HSP60 (1:600, ABclonal, Harbin, China), and HSP70 (1:2700, ABclonal, Harbin, China) and β-actin (1:8000, Zenbio, Chengdu, China) were incubated with NC membrane. Then, IgG-HRP (1:8000, Zenbio, Chengdu, China) was incubated. The protein bands were then observed on a grayscale scanner (Gene Gnome XRQ, Cambridge, UK) using an ECL chemiluminescence kit (SparkJade, Harbin, China). The bands were evaluated using Image J (NIH, Bethesda, MD, USA), and the ratio of each target protein’s gray value to that of β-actin was used to express the relative expression of HSPs.

### 2.5. Statistical Analysis

The data were analyzed using SPSS 21.0 software (IBM, Armonk, NY, USA). The normal distribution of the data was examined using the Kolmogorov–Smirnov method. The inter-group and intra-group differences were analyzed using one-way ANOVA. Duncan’s was used for multiple comparisons. The data are presented as mean ± standard deviation (mean ± SD), with significant differences in *p* < 0.05.

## 3. Results

### 3.1. Relative Expression Levels of Immunoglobulins in Ileum Tissue

**Figure 2:**
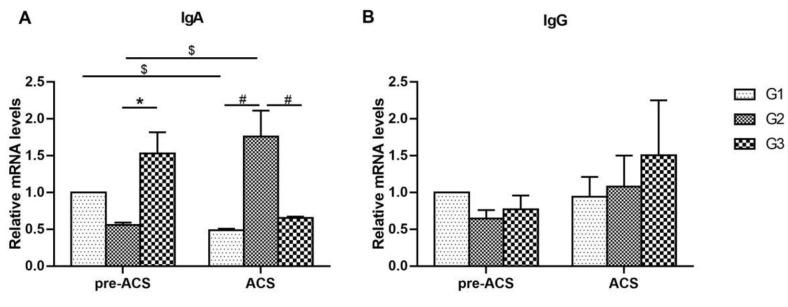
The mRNA levels of immunoglobulins IgA (**A**), and IgG (**B**) in ilea of broilers before and after ACS. Data were presented as mean ± SD (*n* = 6). Different labels represent significant differences (*p* < 0.05). pre-ACS (*), ACS (#) and before and after ACS ($). G1, cold stimulation for 0 h; G2, cold stimulation for 3 h; G3, cold stimulation for 6 h.

Figure 2 shows the mRNA expression levels of immunoglobulins in ilea of broilers before and after ACS. Before ACS, the level of *IgA* expression in the G1 was not vastly different than that in the cold stimulation group (*p* > 0.05). Lower expression level of *IgA* was detected in G2 compared to G3 (*p* < 0.05). Following ACS for 6 h, *IgA* mRNA expression was substantially lower in G1 and G3 than that in G2 (*p* < 0.05). *IgA* expression levels in G1 decreased but significantly increased in G2 after ACS (*p* < 0.05). After ACS, the *IgA* level in G3 did not dramatically change compared with pre-ACS (*p* > 0.05). No obvious difference in the level of *IgG* was observed in all groups before and after ACS (*p* > 0.05).

### 3.2. Relative Expression Levels of Cytokines in Ileum Tissue

**Figure 3:**
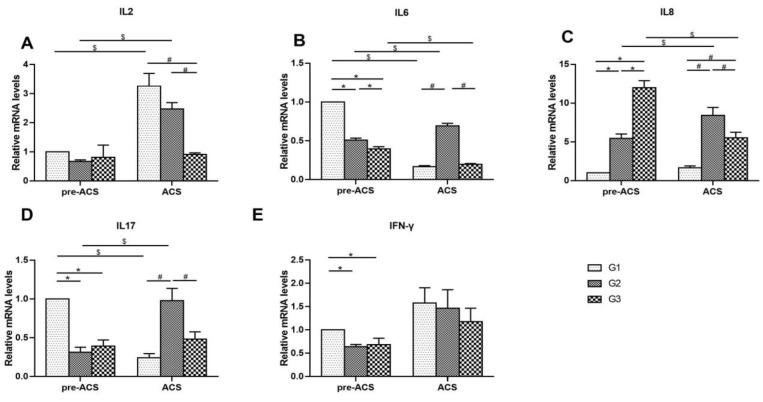
The mRNA levels of cytokines IL2 (**A**), IL6 (**B**), IL8 (**C**), IL17 (**D**), and IFN-γ (**E**) in ilea of broilers before and after ACS. Data were presented as mean ± SD (*n* = 6). Different labels represent significant differences (*p* < 0.05). pre-ACS (*), ACS (#) and before and after ACS ($). G1, cold stimulation for 0 h; G2, cold stimulation for 3 h; G3, cold stimulation for 6 h.

Figure 3 shows the mRNA levels of cytokines in ilea of broilers before and after ACS. Before ACS, the mRNA expression level of IL2 did not differ markedly among all groups (*p* > 0.05). Higher levels of IL6, IL17, and IFN-γ were identified in G1 (*p* < 0.05). The IL8 mRNA expression level was increased dramatically in G2 and G3 compared to G1 and the mRNA expression level of IL8 increased gradually as the cold stimulation time was increased (*p* < 0.05). Following ACS for 6 h, the IL2 mRNA expression level in G3 was found to be down-regulated compared with G1 and G2 (*p* < 0.05), but the IL6, IL8, and IL17 expression levels in G2 compared to G1 and G3 were significantly higher (*p* < 0.05). The IFN-γ level did not differ significantly among treatment groups (*p* > 0.05). The levels of IL2 in G1, as well as IL2, IL6, IL8, and IL17, in G2 were elevated, but the levels of IL6 and IL17 in G1 and IL6 and IL8 in G3 were distinctly lower after ACS (*p* < 0.05). The IFN-γ level did not differ noticeably among treatment groups before and after ACS (*p* > 0.05).

### 3.3. Relative Expression Levels of Toll-Like Receptors in Ileum Tissue

**Figure 4:**
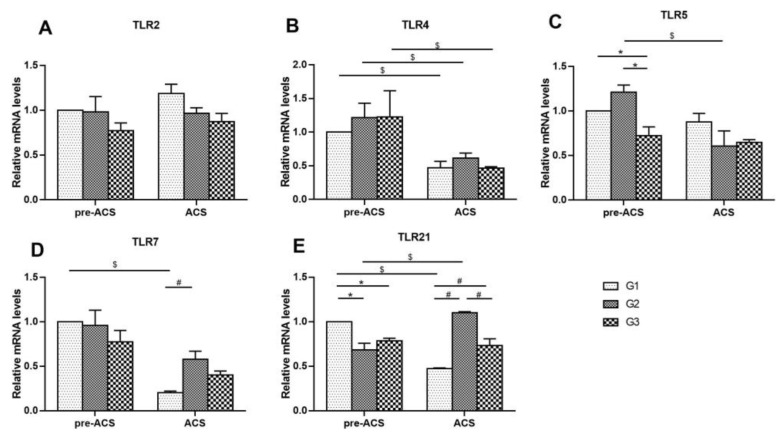
The mRNA levels of Toll-like receptors *TLR2* (**A**), *TLR4* (**B**), *TLR5* (**C**), *TLR7* (**D**), and *TLR21* (**E**) in ilea of broilers before and after ACS. Data were presented as mean ± SD (*n* = 6). Different labels represent significant differences (*p* < 0.05). pre-ACS (*), ACS (#) and before and after ACS ($). G1, cold stimulation for 0 h; G2, cold stimulation for 3 h; G3, cold stimulation for 6 h.

Figure 4 shows the mRNA levels of Toll-like receptors in ilea of broilers before and after ACS. Before ACS, the TLR5 mRNA expression level in G3 was down-regulated compared to G1 and G2 (*p* < 0.05), and the TLR21 mRNA expression in G2 and G3 was down-regulated compared to G1 (*p* < 0.05). TLR2, TLR4, and TLR7 expression levels were not substantially different among all the treatment groups (*p* > 0.05). Following ACS for 6 h, TLR7 mRNA levels were extremely higher in G2 than in G1 (*p* < 0.05), but no visible differences in TLR2, TLR4, and TLR5 mRNA levels were found among all groups (*p* > 0.05). The mRNA level of TLR21 in G2 showed a marked increase compared to G1 and G3 (*p* < 0.05). The expression levels of TLR4, TLR7, and TLR21 in G1 and TLR4, and TLR5 in G2, as well as TLR4 in G3, were dramatically reduced after ACS compared to pre-ACS (*p* < 0.05). There were no differences in TLR2, TLR5, TLR7, and TLR21 in G3 before and after ACS (*p* > 0.05).

### 3.4. Relative Expression Levels of Intestinal Barrier Genes in Ileum Tissue

**Figure 5:**
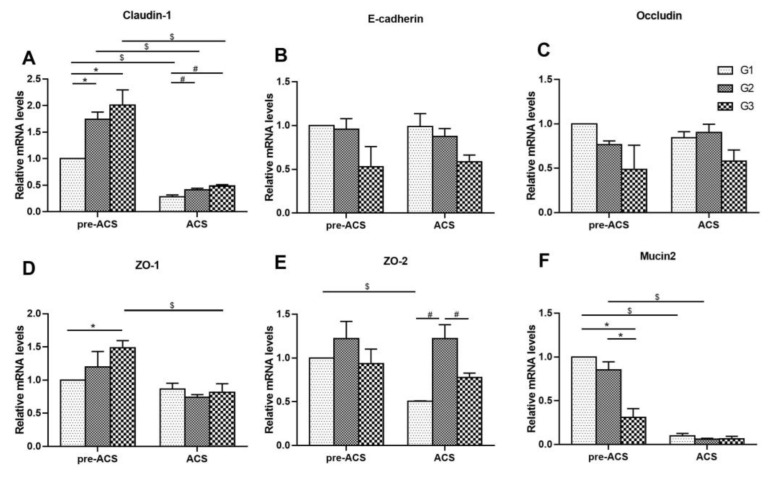
The mRNA levels of intestinal barrier genes *Claudin-1* (**A**), *E-cadherin* (**B**), *Occludin* (**C**), *ZO-1* (**D**), *ZO-2* (**E**), and *Mucin2* (**F**) in ilea of broilers before and after ACS. Data were presented as mean ± SD (*n* = 6). Different labels represent significant differences (*p* < 0.05). pre-ACS (*), ACS (#) and before and after ACS ($). G1, cold stimulation for 0 h; G2, cold stimulation for 3 h; G3, cold stimulation for 6 h.

Figure 5 shows the mRNA levels of intestinal barrier genes in ilea of broilers before and after ACS. Before ACS, the mRNA level of Claudin-1 was dramatically up-regulated in G2 and G3 compared to G1 (*p* < 0.05). The expression level of Mucin2 in G3 was decreased compared to G1 and G2 (*p* < 0.05). The ZO-1 level in G3 was vastly higher than that in G1. The E-cadherin, ZO-2, and Occludin mRNA levels did not noticeably change among all the groups (*p* > 0.05). Following ACS for 6 h, the Claudin-1 mRNA level in G1 was lowest (*p* < 0.05) and the ZO-2 mRNA level was highest in G2 (*p* < 0.05). The E-cadherin, ZO-1, Occludin, and Mucin2 mRNA levels did not differ among all the groups (*p* > 0.05). Lower levels of Claudin-1, Occludin, ZO-2, and Mucin2 in G1 and Claudin-1 and Mucin2 in G2 were detected after ACS (*p* < 0.05), and the Claudin-1, ZO-1 levels in G3 were visibly lower after ACS (*p* < 0.05). The E-cadherin, Occludin, ZO-2, and Mucin2 levels in G3 did not markedly differ before and after ACS (*p* > 0.05).

### 3.5. Relative Expression Levels of Heat Shock Proteins in Ileum Tissue

**Figure 6:**
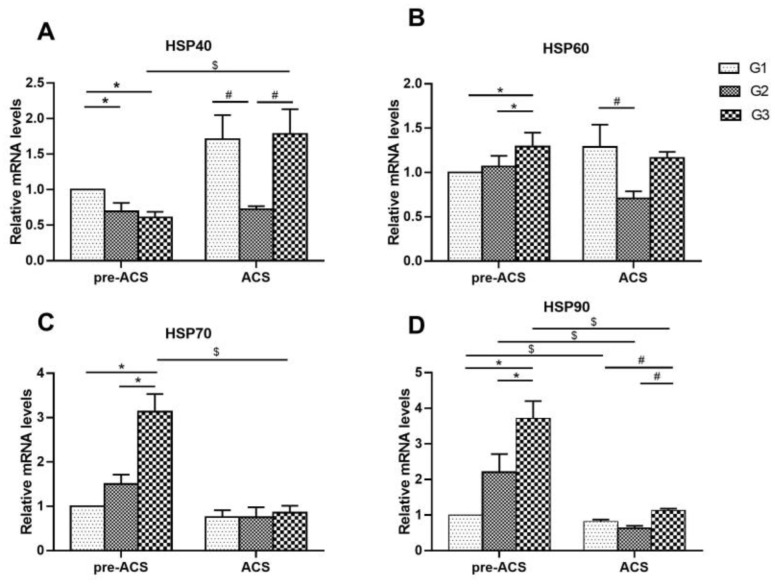
The mRNA levels of heat shock proteins *HSP40* (**A**), *HSP60* (**B**), *HSP70* (**C**), and *HSP90* (**D**) in ilea of broilers before and after ACS. Data were presented as mean ± SD (*n* = 6). Different labels represent significant differences (*p* < 0.05). pre-ACS (*), ACS (#) and before and after ACS ($). G1, cold stimulation for 0 h; G2, cold stimulation for 3 h; G3, cold stimulation for 6 h.

**Figure 7:**
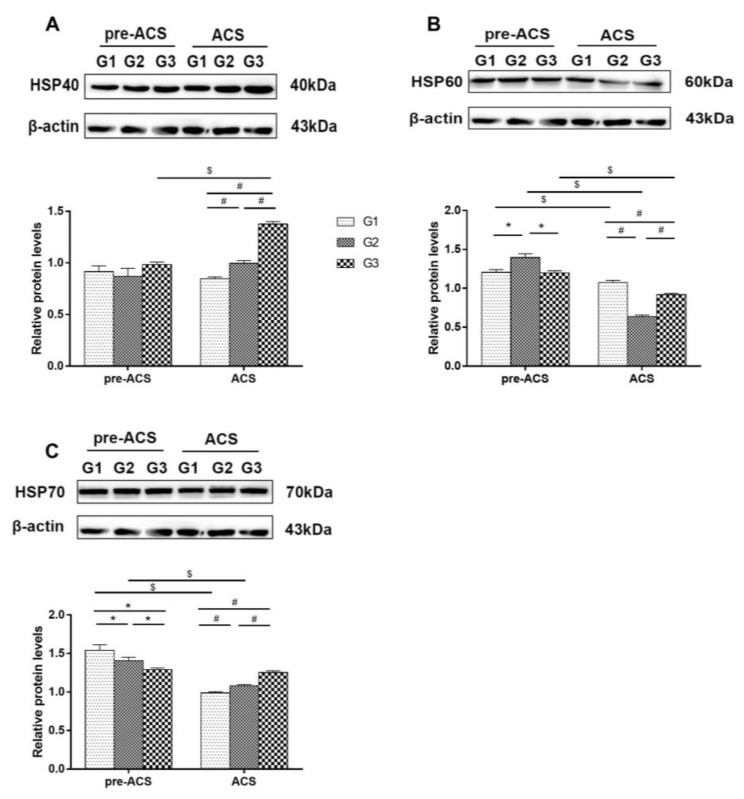
The protein levels of heat shock proteins *HSP40* (**A**), *HSP60* (**B**), and *HSP70* (**C**) in ilea of broilers before and after ACS. Data were presented as mean ± SD (*n* = 6). Different labels represent significant differences (*p* < 0.05). pre-ACS (*), ACS (#) and before and after ACS ($). G1, cold stimulation for 0 h; G2, cold stimulation for 3 h; G3, cold stimulation for 6 h. The original protein images of *HSP40*, *HSP60*, *HSP70*, and *β-actin* are shown in Figures S1–S4.

Figure 6 and Figure 7 show relative levels of heat shock proteins in ilea of broilers before and after ACS. Before ACS, *HSP40* mRNA and *HSP70* protein levels were highest in G1 (*p* < 0.05), and mRNA levels of *HSP60*, *HSP70*, and *HSP90* were considerably elevated in G3 compared to G1 and G2, respectively (*p* < 0.05). The *HSP60* protein levels in G2 were markedly higher than in G1 and G3 (*p* < 0.05). Following ACS for 6 h, a higher level of *HSP90* mRNA in G3 compared to G1 and G2 was observed (*p* < 0.05) and the protein levels of *HSP40* and *HSP70* were vastly up-regulated in G3 compared to G1 and G2 (*p* < 0.05). The mRNA level of *HSP40* in G2 was noticeably down-regulated compared to G1 and G3 (*p* < 0.05), and the mRNA level of *HSP60* in G2 was markedly lower compared to G1 (*p* < 0.05). The expression level of *HSP60* protein in G1 was highest (*p* < 0.05), but no vast difference in *HSP70* mRNA level was observed among the groups (*p* > 0.05). *HSP90* mRNA and *HSP60* protein in each group were decreased significantly after ACS (*p* < 0.05), while *HSP40* mRNA and *HSP40* protein level in G3 showed an increasing trend, but *HSP70* mRNA levels were dramatically down-regulated after ACS (*p* < 0.05). Finally, the levels of *HSP70* protein in G1 and G2 decreased significantly after ACS (*p* < 0.05).

## 4. Discussion

Low temperatures have a serious effect on the development of animal husbandry. It is generally believed that an adverse environment, such as one that is cold, will have a negative impact on immune function [19,46,47] and cause inflammation or inflammatory diseases [5,48]. However, it has been demonstrated that appropriate cold stimulation training during the early growth stage of animals can make them adapt to a cold environment and improve their immune function and anti-stress ability [7,8]. Thaxton et al. [49] proved that exposing broilers to a cold environment can improve the synthetic rate of *IgA* and enhance humoral immunity. The aim of this research was to explore the effects of early intermittent cold stimulation training on ileum immune system and anti-stress capacity of broilers subjected to acute cold stress.

Immunoglobulins are crucial to the body’s self-defense; their expression levels can reflect the body’s resistance to disease as well as its immune status [50]. Cold stress can promote immunoglobulins expression levels and, thus, exert a protective effect on the intestine [7]. Zhao et al. [48] demonstrated that after exposing broilers to a low temperature for a period of time, *IgA* and *IgG* gene expression levels noticeably up-regulated in the duodenum and jejunum, and intestinal immune function was enhanced. Carr et al. [51] proved a vastly up-regulate in *IgA* mRNA expression in the intestinal tissues of mice (*Mus musculus*) exposed to −20 °C for nine consecutive days (20 min each time). Appropriate cold stimulation can, thus, increase the immunoglobulins expression levels and strengthen the body’s immune ability and stress resistance. The current study revealed that after ACS, the level of *IgA* in the ileum of broilers in G1 were dramatically down-regulated, which indicates that ACS could damage broilers’ immune systems. However, *IgA* levels in G2 trained with cold stimulation were significantly increased when subjected to ACS, suggesting that the broilers need to up-regulate immunoglobulin level to protect the intestine. The G3 group have already established cold adaptation, did not need to produce too many immunoglobulins to relieve ACS (10 °C). Similar results were found that immunomodulatory and antioxidative functions of broilers can be improved when broilers are kept at 3 °C lower than ambient temperature for a long time before being subjected to acute cold stress [20,21]. These results further demonstrate that moderate cold stimulation in early life can increase the expression levels of immunoglobulins, and relieve the harm caused by ACS at a later stage.

Cytokines are absolutely necessary for the two-way communication between the immune and endocrine systems [52,53]. Th1 cells produce *IFN-γ* and *IL-2*, which are responsible for cellular immunity, whereas Th2 cells produce *IL-4* and *IL-10*, which are responsible for humoral immunity [54]. Cold stimulation can affect the body’s immune system by affecting the content of cytokines. Zhao et al. [48] found that exposure of broilers to 12 °C for 21 days significantly reduced *IFN-γ* but *IL4* significantly increased in their ileum, leading to an immune imbalance. In the present study, after acute cold stimulation, the level of *IL2* in broilers of G1 without cold stimulation training was significantly increased, which suggested that broilers were affected by cold stress and responded to the low temperature by improving cellular immunity. A similar result was reported that the level of *IL2* in the serum of Wistar rats was significantly increased after three days/4 °C cold stress [55]. The levels of *IL2* in G3 showed no significant difference after ACS, indicating that broilers had adapted to the cold environment due to early cold training. According to the study’s findings, ACS considerably increased the *IL6*, *IL8*, and *IL17* level in the G2 while dramatically lowering *IL8* level in the G3 group, indicating that environmental changes of this nature will have an adverse impact on G2 group, and induce the body to produce pro-inflammatory factors. However, the G3 group was better adapted to the cold environment after early cold stimulation training, which is not needed to aggregate pro-inflammatory factors and cause an inflammatory response. Brenner demonstrated that plasma *IL6* expression levels were increased after exposure to a cold environment [56]. Monroy’s study showed that cold water stress in mice for five minutes a day led to an increase in *IFN-γ* and *IL6* protein levels [57]. It can be concluded that broilers with established cold adaptation will inhibit the release of proinflammatory cytokines when suffering from ACS at 10 °C. The anti-stress ability of broilers can be improved after suitable cold stimulation training at an early stage of life.

Pattern-recognition receptors known as toll-like receptors (TLRs) are a principal mediator of the innate immune response [58]. TLRs are involved in the acute response phase of the body [59]. TLRs can cooperate with each other to promote the immune response [28,60]. *TLR4*, *TLR7*, and *TLR21* expression levels in G1 broilers without cold stimulation training were considerably down-regulated after ACS, indicating that ACS leads to immune dysfunction in broilers. Quinteiro-Filho reported that broilers receiving chronic heat stimulation for 10 h every day until six days before being sampled could decrease the amount of *TLR4* expression in their spleens and impair their immune function [61]. However, the *TLR2*, *TLR5*, *TLR7*, and *TLR21* levels in broilers of G3 trained with cold stimulation were not significantly different before and after ACS, indicating that the early cold stimulation training made broilers adapt to cold environments and improve cold resistance. In the present study, after ACS, the level of *TLR5* dropped considerably in broilers of G2, the reason may be that the broilers had not yet established cold adaptation. The result was consistent with Basu. Basu et al. [62] who showed that *TLR5* in Indian major carp catla (*Catla catla*) significantly decreased after acute cold stress. This indicates that the body is not strong enough to resist ACS. Broilers of G2 would mobilize *TLR21* to resist cold stress after ACS. *TLR21* level in G3 was not vastly difference before and after ACS, indicating that acute cold stress was within the range of adjustment of the body, and early cold training enhanced stress resistance.

The intestinal barrier separates the enteric cavity of the intestine from the internal environment of the organism. *Occludin* and *Claudins* form the tight junction backbone [41]. Junction adhesion molecules are valuable in immune cells transfer during immune surveillance and the inflammatory response [63]. In our investigation, the *Occludin* and *ZO-2* mRNA levels in broilers of G1 were dramatically down-regulated after ACS, which was in line with He’s discovery. He et al. [28] proved that heat stress for three days could reduce the protein levels of *Occludin* and *ZO-2* in the small intestine of rats (*Rattus norvegicus*). However, the *Occludin* expression level in G2 and G3 groups did not noticeably change before and after ACS. Based on the study’s findings, when broilers of G1 without cold stimulation training are exposed to ACS, intestinal permeability increases and tight junction gene expression levels in the ileum significantly decrease, whereas broilers of G3 can adapt to a cold environment and resist the harm caused by ACS. The expression of *Mucin2* dropped considerably after ACS in G1 and G2, similar results were found in the research of Zhang [37]. The above results indicate that cold stress has adverse effects on the intestinal tract and inhibits the production of *Mucin2.* However, the *Mucin2* level did not dramatically change in G3 before and after ACS, which may be because the broilers experienced an improved ability to resist cold stress in the early stage of cold training, so as to activate the body’s protective mechanism to prevent bacteria from invading the intestinal wall. In conclusion, broilers without established cold adaptation will have down-regulated intestinal barrier gene levels and increased intestinal permeability when subjected to ACS, while cold stimulation training for six hours at one day intervals can lead to an ability to resist the intestinal damage caused by acute cold stress at a later stage.

Heat shock proteins (HSPs) exert a protective effect on cells [64,65]. When cells are stimulated by stress, they will induce the synthesis of HSPs, which participate in cell protective functions and increase the tolerance of cells to stress. Xu et al. [44] found that *HSP27* and *HSP70* levels were dramatically elevated in porcine cardiomyocytes under transport stress, and this increase in expression was accompanied by a reduction in myocardial injury, so this may be associated with the protection of cardiomyocytes. Wei et al. [20] confirmed that 24 h of acute cold stress in broilers after 34 days of chronic cold stimulation markedly up-regulated the expression of *HSP40* in the heart. In the current research, the G3 group’s levels of *HSP40* protein and mRNA considerably increased after ACS compared to pre-ACS, which is consistent with the results of Wei’s study, suggesting that the body alleviates the damage caused by stress by up-regulating the expression of *HSP40*. *HSP70* and *HSP90* are vital to prevent damage to intestinal epithelial and mucosal cells [66]. In this study, the HSPs (*HSP60*, *HSP70*, *HSP90*) levels in G3 substantially higher than those of in G1 during early cold stimulation, indicating that cold stress will modulate HSPs levels, thus protecting the intestinal mucosal barrier [67] and helping the body adapt to the environment. Puijvelde et al. [68] showed that the high levels of HSPs under the same stimulation represent a potent capacity for stress resistance. The G1 and G2 groups in this study had considerably lower levels of *HSP90* mRNA, *HSP40* protein, and *HSP70* protein after ACS than in the G3 group, indicating that the G1 and G2 groups had a weak ability to resist cold stress. It can, thus, be seen that broilers trained by 6 h of cold stimulation have improved anti-stress abilities and can swiftly activate the expression of HSPs when exposed to ACS.

## 5. Conclusions

The findings of the present research show that cold stimulation training with an interval of one day, at 3 °C below the conventional temperature can change the immune function and improve cold resistance, and that the intestinal damage can be relieved when subjected to ACS in the later stage. Moreover, the six-hour intermittent cold stimulation scheme makes broilers more resistant to cold stress.

## Data Availability

Not applicable.

## Supplementary Materials

The following supporting information can be downloaded at: https://www.mdpi.com/article/10.3390/ani12233260/s1, Figure S1: The original protein image of *HSP40*; Figure S2: The original protein image of *HSP60*; Figure S3: The original protein image of *HSP70*; Figure S4: The original protein image of *β-actin*; Table S1: Primer sequences used for the study.
