# Supplementary material for: Effects of Acute Cold Stress after Intermittent Cold Stimulation on Immune-Related Molecules, Intestinal Barrier Genes, and Heat Shock Proteins in Broiler Ileum
Source: Animals (Basel). 2022 Nov 23;12(23):3260. doi: 10.3390/ani12233260 (PMC9739716; doi:10.3390/ani12233260)
Supplement: Supplementary file 1 [file animals-12-03260-s001.zip › animals-1957859-supplementary.pdf]

Table S1. Primer sequences used for the study

	Reference sequence	Primer sequence(5′-3′)
β-actin	NM_205518.1	F: CACCACAGCCGAGAGAGAAAT
		R: TGACCATCAGGGAGTTCATAGC
IgA	NM_205287.1	F: TGCTAGTGGTTGTGGTGCTTGTG
		R: CGGAGGCGGAGGAGACGATG
IgG	XM_025146241.1	F: CGATTCCAGCCTCAGCGTCAC
		R: TAGGTGCCGTTGAAGTGTTCTTGG
IFN-γ	NM_205149.1	F: GAACTGGACAGGGAGAAATGAGA
		R: ACGCCATCAGGAAGGTTGTT
IL-2	NM_204153.1	F: CTGTATTTCGGTAGCAATG
		R: ACTCCTGGGTCTCAGTTG
IL-6	NM_204628.1	F: AAATCCCTCCTCGCCAATCT
		R: CCCTCACGGTCTTCTCCATAAA
IL-8	NM_205018.1	F: GGCTTGCTAGGGGAAATGA
		R: AGCTGACTCTGACTAGGAAACTGT
IL-17	NM_204460.1	F: GCCATTCCAGGTGCGTGAACTC
		R: CGGCGGAGGACGAGGATCTC
TLR2	XM_001232192	F: GATTGTGGACAACATCATTGACTC
		R: AGAGCTGCTTTCAAGTTTTCCC
TLR4	NM_001030693.1	F: AGTCTGAAATTGCTGAGCTCAAAT
		R: GCGACGTTAAGCCATGGAAG
TLR5	NM_001024586	F: CCTTGTGCTTTGAGGAACGAGA
		R: CACCCATCTTTGAGAAACTGCC
TLR7	NM_001011688	F: TTCTGGCCACAGATGTGACC
		R: CCTTCAACTTGGCAGTGCAG
TLR21	NM_001030558	F: TGCCCCTCCCACTGCTGTCCACT
		R: AAAGGTGCCTTGACATCCT
Claudin-1	NM_001013611.2	F: TGGAGGATGACCAGGTGAAGA
		R: CGAGCCACTCTGTTGCCATA
E-cadherin	NM 001039258.2	F: GACAGGGACATGAGGCAGAA
		R: GCCGTGACAATGCCATTCTC
Occludin	NM 205128.1	F: TCATCGCCTCCATCGTCTAC
		R: TCTTACTGCGCGTCTTCTGG
ZO-1	XM 413773.4	F: TGTAGCCACAGCAAGAGGTG
		R: CTGGAATGGCTCCTTGTGGT
ZO-2	XM_025144669.1	F： CGGCAGCTATCAGACCACTC
		R： CACAGACCAGCAAGCCTACAG
Mucin2	XM 421035	F: CAGCACCAACTTCTCAGTTC
		R: TCTGCAGCCACACATTCTTT
HSP40	NM_001199325.1	F: GGGCATTCAACAGCATAGA
		R: TTCACATCCCCAAGTTTAGG
HSP60	NM_001012916.2	F: AGCCAAAGGGCAGAAATG

		R: TACAGCAACAACCTGAAGACC
HSP70	NM_001006685.1	F: CGGGCAAGTTTGACCTAA
		R: TTGGCTCCCACCCTATCTCT
HSP90	NM_001109785.1	F: TCCTGTCCTGGCTTTAGTTT
		R: AGGTGGCATCTCCTCGGT

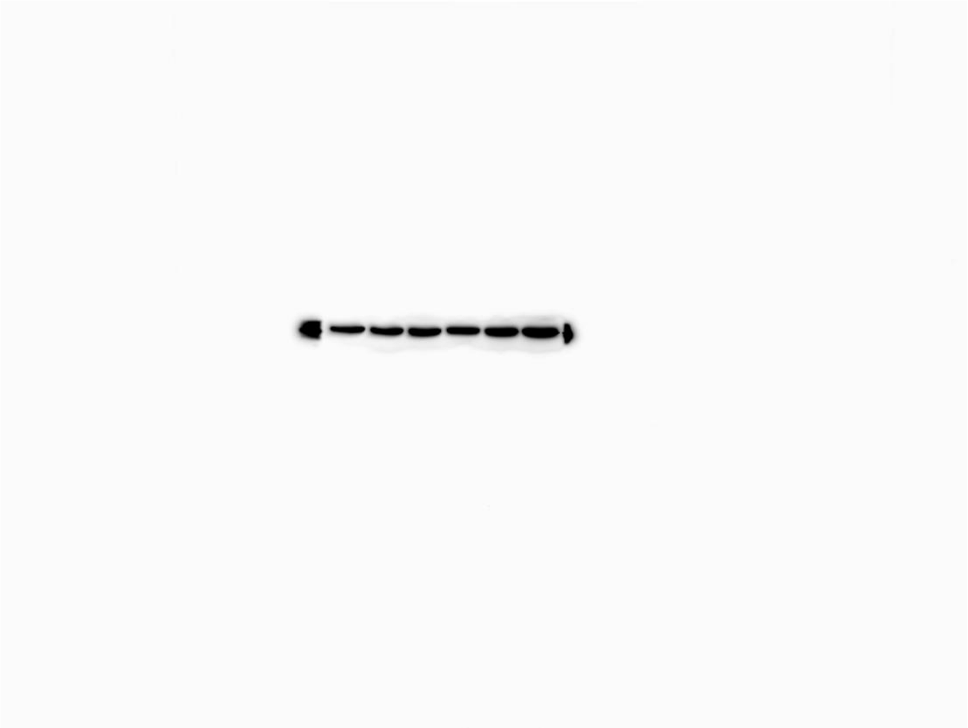

Figure S1. The original protein image of HSP40. The lanes from left to right represent: G1 (pre-ACS), G2 (pre-ACS), G3 (pre-ACS), G1 (ACS), G2 (ACS), G3 (ACS).

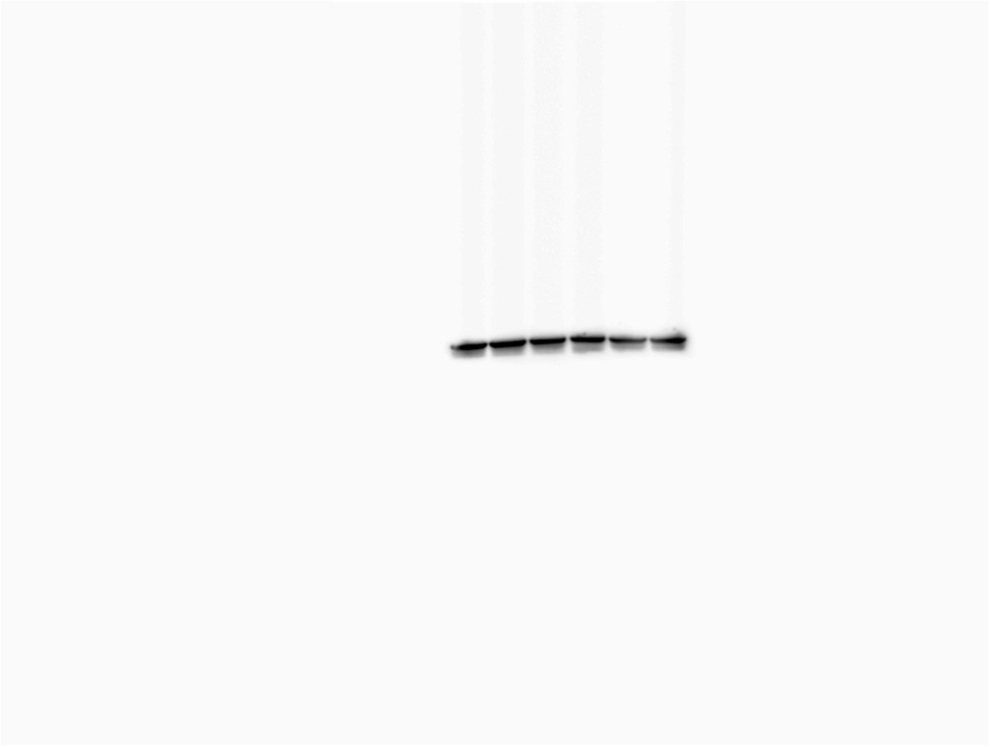

Figure S2. The original protein image of HSP60. The lanes from left to right represent: G1 (pre-ACS), G2 (pre-ACS), G3 (pre-ACS), G1 (ACS), G2 (ACS), G3 (ACS).

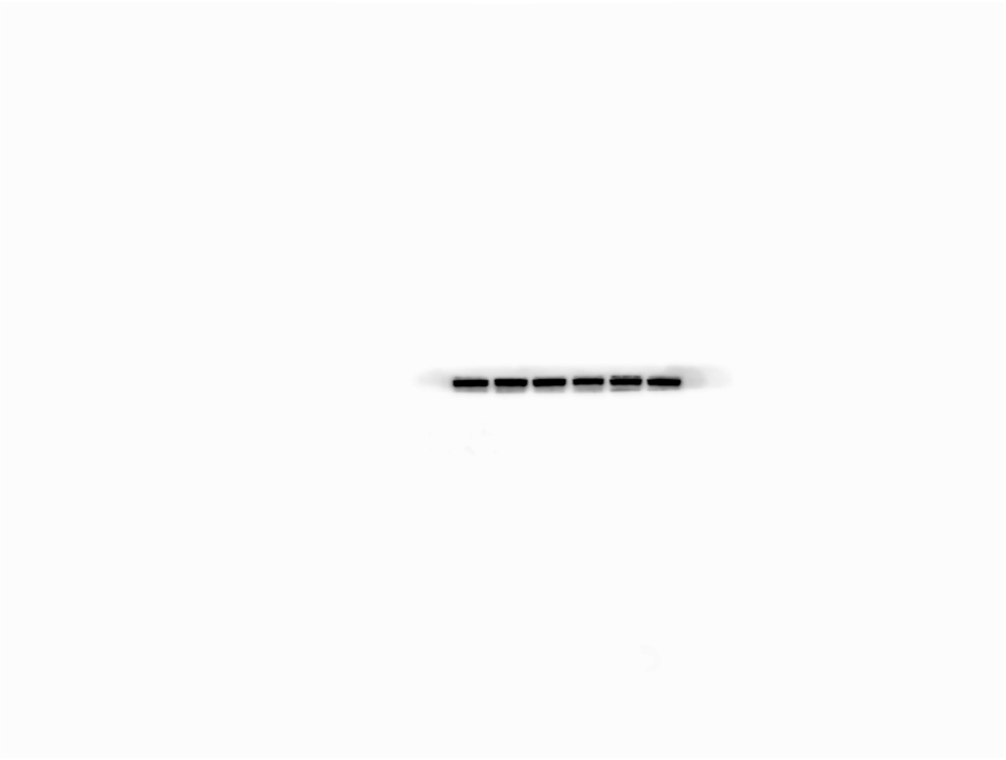

Figure S3. The original protein image of HSP70. The lanes from left to right represent: G1 (pre-ACS), G2 (pre-ACS), G3 (pre-ACS), G1 (ACS), G2 (ACS), G3 (ACS).

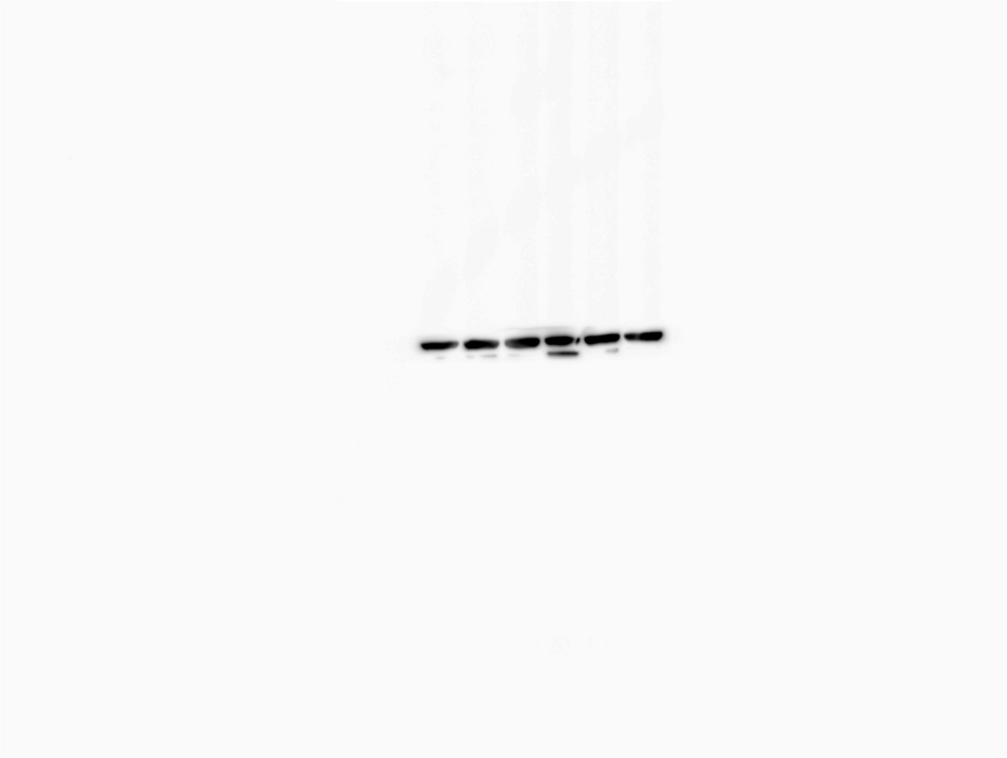

Figure S4. The original protein image of β-actin. The lanes from left to right represent: G1 (pre-ACS), G2 (pre-ACS), G3 (pre-ACS), G1 (ACS), G2 (ACS), G3 (ACS).
